# Supplementary material for: The predictive value of the dynamic change slope of red blood cell distribution width to platelet ratio combined with clinical indicators for the mortality outcome of patients with sepsis
Source: PLoS One. 2026 Mar 2;21(3):e0343684. doi: 10.1371/journal.pone.0343684 (PMC12952655; doi:10.1371/journal.pone.0343684)
Supplement: Attachment — Submitted filename: Response to Reviewers.pdf [file pone.0343684.s003.pdf]

Response to Reviewers

Manuscript ID: PONE-D-25-35502
Title: The predictive value of the dynamic change slope of red blood cell distribution width to platelet ratio combined with clinical indicators for the mortality outcome of patients with sepsis

Dear Editor and Reviewer,

We sincerely thank the Academic Editor and the reviewer for the constructive and insightful comments. We have revised the manuscript thoroughly according to all suggestions. Below we provide a point-by-point response. All modifications have been incorporated into the revised manuscript, and changes are marked accordingly. (Line numbers refer to the clean revised manuscript, and changes are highlighted in the marked version)

Reviewer 1 Point-by-Point Responses

Comment 1.Lines 41-45 Reference needed for number

Response

Thank you to the reviewers for pointing out the errors. The original manuscript's statement about "mortality rate as high as 18.7%" was not precise enough—this figure comes from Bauer et al. (2020) reporting a 30-day sepsis pooled mortality rate of 18.7% in their systematic review of Australia (Bauer M. Crit Care 2020), rather than referring to all hospitalized sepsis patients.

Amendments

Considering the inaccuracy of this data, after reviewing the literature, We cited a broader pooled data and revised the estimated mortality rate for hospitalized sepsis patients and the pre-discharge mortality rate for sepsis patients receiving intensive care treatment. Data from Fleischmann-Struzek et al., Intensive Care Med 2020, indicates that the pooled hospital mortality rate for hospital-treated sepsis is approximately 26.7% (95% CI 22.9–30.7), while the pre-discharge mortality rate for sepsis patients treated in the intensive care unit is as high as approximately 41.9% (95% CI 36.2–47.7) . We have added the reference in the revised manuscript (**Introduction, first paragraph, lines 41-45**).

Comment 2 (Lines 46) – What is “slow immunity”？

Response

We agree that the expression “slow immunity” is inaccurate and unclear. It has been rewritten to describe the intended meaning more precisely.

Amendments

Revised to “**impaired immune function and compromised host defenses**” in the **Introduction** (**second paragraph, lines 46**).

Comment 3 (Lines 52-54)- Reference needed

Response

Relevant references have been added to support this statement.

Amendments

New citation has been inserted in the revised manuscript (**Introduction, third paragraph, lines 54**).

Comment 4 (Lines 70) – what diagnosis of sepsis was used? When was the diagnosis sepsis taken? On admission? Or generally during the ICU-stay?

Response:

We appreciate this comment. We now clearly describe that sepsis was diagnosed according to the Sepsis-3 criteria, and the diagnosis was made at the time of ICU admission.

Amendments:

Clarification added in the **Research Methods(lines 70)**: “Sepsis was diagnosed according to the Sepsis-3 definition (increase in SOFA ≥2) at the time of ICU admission.”

Comment 5 (Lines 73) - Please elaborate further, as this contradicts the inclusion criterium of sepsis/organ dysfunction

Response:

Thank you for pointing out the potential inconsistency between the Sepsis-3 definition, which requires organ dysfunction (SOFA ≥ 2), and our exclusion criterion

describing “severe organ dysfunction”. We agree that the original wording could cause misunderstanding. Our intention was not to exclude patients with sepsis-related organ dysfunction defined by SOFA, but to exclude patients with pre-existing, end-stage, irreversible organ failure that was unrelated to the current sepsis episode and indicated imminent death.

Amendments:

To avoid confusion, we have revised the **exclusion criterion** (**Research methods,first paragraph, lines 73-76**) to:

“Existing end-stage or irreversible organ failure unrelated to current sepsis (e.g., advanced heart failure, end-stage renal disease requiring long-term dialysis, end-stage liver disease, or long-term ventilator dependence).”

We believe this modification clarifies the criterion and is consistent with the Sepsis-3 diagnostic definition.

Comment 6 (Lines 82) – How was written consent obtained from dead patients? Or was it obtained from all patients admitted with sepsis? When was it obtained? And what about sedated patients or patients incapable to provide consent. Please revise wording.

Response:

We apologize for the unclear description.

We now clarify that:

We obtain written informed consent from all conscious and mobile sepsis patients admitted to the ICU;

For comatose, sedated, or unable-to-sign informed consent forms, their family members or legal guardians sign the forms;

For patients who subsequently die, their family members or guardians have already signed informed consent forms upon their admission to the ICU.

Amendments:

The ethical statement in the revised manuscript (**Research Methods, second paragraph, lines 82-87**) has been rewritten to:

“Written informed consent was obtained from patients or their legally authorized representatives at ICU admission. For patients who were unconscious or unable to sign, consent was provided by family members or legal guardians.”

Comment 7 (Lines 146-157) – Were all patients compared or only

subsets including missing data? See also example below. Survivors only stayed 3 days (mean) – how was data handled after discharge from ICU. Alternatively, this is a limitation of the analysis and conclusion.

Response:

Thank you for this important comment. All patients included in the final analysis had complete RPR-related laboratory measurements for the first 5 days after ICU admission. Patients with missing RPR data on any day within this 5-day window were excluded prior to analysis.

At our institution, routine hematological testing is typically performed during the early phase of critical illness, including after transfer from the ICU to the general ward when clinically indicated. Therefore, RPR measurements were generally available even for survivors with shorter ICU stays.

Amendments:

We have clarified this process in the revised **Research Methods** (Data collection **lines 100-107** and Statistical analysis **lines 121-123**) and **Results** (Comparison of clinical data, **lines 154-156**) section and acknowledged in the **Discussion** (limitations **lines 358-363**) that restricting the analysis to a 5-day window was a design choice to minimize missing data, while longer observation periods might increase attrition and potential selection bias. We hope this revision adequately addresses the reviewer's concern.

Comment 8 (Lines 158) – Table 1: ICU stay/d: ICU stay of a mean of 3 days in the survivors group? Did blood collections occur until day 5 then? Or were these days considered as missings in the statistical analysis.

Response:

Thank you for your valuable feedback. We sincerely apologize for the initial lack of clarity in explaining the data collection process.

Yes, the mean ICU stay in the survivors group was approximately 3 days. However, laboratory data collection was anchored to ICU admission rather than ICU stay duration. Routine blood testing continued during the early post-ICU period when clinically indicated, allowing RPR measurements to be obtained for up to 5 days after

ICU admission.

Patients with incomplete RPR measurements within this 5-day period were excluded from the final analysis, and no imputation was performed. We have clarified this point in the Methods section.

Amendments:

We have clarified this in the revised **Research Methods(lines 100-107)** section to avoid misunderstanding.

Comment 9. Surgical and non-surgical patients with sepsis might differ in outcome. Can the authors provide numbers on the admissions of surgical and non-surgical patients. Did these groups differ in outcome?

Response:

Thank you for this important comment. We agree that outcomes may differ between surgical and non-surgical patients with sepsis. However, in this retrospective study, the indication for ICU admission (surgical vs. non-surgical) was not consistently or reliably recorded in the electronic medical records, and therefore this variable was not systematically collected for analysis.

As a result, we are unable to provide accurate subgroup numbers or perform a reliable outcome comparison between surgical and non-surgical patients. We have acknowledged this **limitation (lines 372-376)** in the revised **Discussion** section, noting that surgical status may represent a potential confounding factor and should be addressed in future prospective studies with more detailed clinical stratification.

Comment 10 (Lines 188) – Please add *P* values to the Table

Response:

We thank the reviewer for this suggestion. *P* values for the estimated marginal means were obtained using Wald tests based on the generalized estimating equation (GEE) model. Specifically, pairwise comparisons between groups at each time point were conducted using model-based contrasts.

Amendments:

We have added the corresponding ***P* values** to the **table 2(lines 187-189)** and clarified the **Statistical analysis** (second paragraph, **lines 125-127**) accordingly.

Comment 11 (Lines 224-225) - septic shock and positive microbial probes were also significantly different between the groups, why were they not included in the Multivariate logistic regression analysis?

Response:

Thank you for this comment.

We agree that several variables, including septic shock, positive microbial culture, APACHE II score, and others, were significantly associated with mortality in univariate analyses.

However, given the limited number of outcome events (*n* = 37), the number of variables entered into the multivariate logistic regression model was restricted to avoid overfitting. Variables were therefore selected based on clinical relevance, biological plausibility, and avoidance of collinearity.

Second, APACHE II and septic shock are closely associated with indicators of disease severity, such as SOFA score, lactate level, and the need for mechanical ventilation. Including septic shock alongside these variables in the model introduces significant multicollinearity and may impair model stability and interpretability.

Third, although univariate analysis showed that positive microbial culture results were associated with mortality, they are not direct indicators of disease severity and may be affected by sampling time, prior antibiotic exposure, and test sensitivity. Therefore, they were not considered suitable independent predictors for inclusion in a multivariate model.

The RPR slope was included in the model as the primary variable of interest. SOFA score and lactate level represent the severity of organ dysfunction and tissue hypoperfusion, respectively. Mechanical ventilation was included in the model as an indicator of severe respiratory failure and treatment intensity.

In the initial exploratory multivariate analysis, comorbidities were included together with other candidate variables. However, comorbidities did not show a significant association with mortality in univariate analysis and demonstrated limited stability in the multivariate model, with a wide confidence interval.Therefore, comorbidities were excluded from the final model.

Based on these considerations, we ultimately selected clinically meaningful and non-redundant variables (RPR slope, lactate level, SOFA score, and mechanical ventilation time) to construct a multivariate logistic regression model. The rationale for this selection has been explained in the revised draft.

Amendments:

We have revised the **Results** of the **logistic regression analysis** in **Table 3(lines 209-223)** and clarified the **Statistical analysis** (third paragraph, **lines 137-140**) accordingly. We hope this revision adequately addresses the reviewer's concern.

Comment 12 (Lines 254) – besides the physiology and mechanistic relevance of RDW a discussion of clinical applications would help the reader to evaluate the relevance of RDW in clinical practice. Lately, RDW has been investigated in studies on critically ill patients with e.g. ARDS, renal failure, or pancreatitis

Response:

We thank the reviewers for their valuable suggestions. In the revised discussion section, we have added a new paragraph exploring the clinical value of RDW in critically ill patients. Specifically, we now discuss the application of RDW as an readily available and cost-effective biomarker in prognostic assessment and risk stratification of various critical illnesses, including acute respiratory distress syndrome, acute kidney injury, and other forms of critical illness. This addition helps to place our findings in a broader clinical context and highlights the practical value of RDW in the intensive care setting.

Amendments:

We have added a section to the **discussion section (second paragraph, lines 268-278)** on the clinical value of RDW in critically ill patients.

Comment 13 (Lines 351) - Limitations section should be expanded a bit more. Especially survivors stayed short on the ICU. Maybe they were not sick enough?

Response:

We thank the reviewer for this important comment. We agree that the shorter ICU stay observed in survivors may reflect a lower baseline severity of illness, which could influence the interpretation of dynamic RPR changes.

Accordingly, we have expanded the limitations section to explicitly acknowledge that survivors tended to have shorter ICU stays and that differences in RPR dynamics between survivors and non-survivors may partially reflect underlying disease severity rather than a purely independent prognostic effect. We have also emphasized that, despite multivariable adjustment, residual confounding cannot be completely excluded.

These additions help to more clearly define the scope and clinical interpretation of our findings.

Amendments:

We have supplemented the **limitations** and shortcomings in the Revised manuscript (Discussion section, **lines 364-371**).

Thank you very much for your attention and time. Look forward to hearing from you.

Your sincerely,
Yun Sun
Jan 06 2026
E-mail: sunyun15@163.com
