# Supplementary material for: The predictive value of the dynamic change slope of red blood cell distribution width to platelet ratio combined with clinical indicators for the mortality outcome of patients with sepsis
Source: PLoS One. 2026 Mar 2;21(3):e0343684. doi: 10.1371/journal.pone.0343684 (PMC12952655; doi:10.1371/journal.pone.0343684)
Supplement: Attachment — Submitted filename: Response_to_Reviewers_auresp_2.pdf [file pone.0343684.s004.pdf]

Response to Reviewers

Manuscript ID: PONE-D-25-35502 R1
Title: The predictive value of the dynamic change slope of red blood cell distribution width to platelet ratio combined with clinical indicators for the mortality outcome of patients with sepsis

Dear Editor and Reviewer

We sincerely thank the Academic Editor and the reviewers for their careful evaluation of our manuscript and for their constructive comments. We have carefully addressed all comments and revised the manuscript accordingly to improve clarity and methodological transparency. All modifications have been incorporated into the revised manuscript, and changes are marked accordingly. Below we provide a point-by-point response. (Line numbers refer to the clean revised manuscript, and changes are highlighted in the marked version)

Reviewer 3 Point-by-Point Responses

Comment 1.Did the author also exclude patients with hemolytic anemia e.g. G6PD deficiency or autoimmune hemolytic anemia? as those may have increased RDW as well. Please clarify.

Response

We thank the reviewer for highlighting this important potential confounder. In our original study design and data screening process, patients with pre-existing hematological disorders, including hemolytic anemia, were excluded from the final analysis. However, this was not explicitly specified in the exclusion criteria in the initial manuscript.

To enhance clarity and methodological transparency, we have revised the Research Methods section to explicitly state that patients with hemolytic anemia, such as glucose-6-phosphate dehydrogenase deficiency and autoimmune hemolytic anemia, were excluded. This ensures that RDW variations related to hemolytic conditions did not influence our findings.

Amendments

The exclusion criteria in the **Research Methods** section(**lines 80-82**) have been revised to explicitly include hemolytic anemia (e.g., G6PD deficiency and autoimmune hemolytic anemia) under pre-existing hematological disorders.

Comment 2.Did the authors exclude the patients who received RBC and platelet transfusion during day 1 to day 5 of admission? as those would affect the RDW and platelet. Please clarify.

Response

We appreciate the reviewer's valuable comment regarding the influence of transfusion on dynamic RDW and platelet measurements. In our original study design and actual data analysis, patients who received red blood cell or platelet transfusions during the first five days of ICU admission were excluded from the final cohort to avoid distortion of laboratory parameters. In addition, patients with any red blood cell or platelet transfusion before ICU admission were also excluded.

However, this was not clearly described in the initial exclusion criteria. Therefore, we have now explicitly clarified this in the Research Methods section to improve transparency.

Amendments

The **Research Methods** section(**lines 84-86**) has been updated to specify exclusion of patients who received any red blood cell or platelet transfusion before ICU admission or within the first five days of ICU hospitalization.

Journal Requirements – References

Response:

We have thoroughly checked all references for accuracy, completeness, and retraction status in accordance with the journal's requirements. No retracted articles were identified and no modifications were required.

Closing Statement

We sincerely appreciate the Academic Editor and reviewers for their thoughtful comments, which have helped us improve the clarity and rigor of our manuscript. We hope that the revised manuscript is now suitable for publication in PLOS ONE.

Thank you very much for your attention and time. Look forward to hearing from you.

Your sincerely,
Yun Sun
February 5, 2026
E-mail: sunyun15@163.com
